# Squamous cell carcinoma in situ arising in mature cystic teratoma of the ovary: a case report

**DOI:** 10.1186/1757-2215-4-5

**Published:** 2011-03-24

**Authors:** Fatima A Zakkouri, Saloua Ouaouch, Saber Boutayeb, Mouna Rimani, Lamiae Gamra, Hind Mrabti, Hassan Errihani

**Affiliations:** 1 Department of Medical Oncology, National Institute of Oncology, Rabat, Morocco; 2 Laboratory of Histopathology "Hassan", Rabat, Morocco

## Abstract

**Introduction:**

Malignant transformation is a rare complication of mature cystic teratoma with squamous cell carcinoma being the most common type. We report a new case of squamous cell carcinoma in situ.

**Case presentation:**

A 62 year old woman was admitted for an abdomino-pelvic mass and she underwent a left salpingo-oophorectomy. The histopathologic analysis revealed a squamous cell carcinoma in situ arising in mature cystic teratoma of the ovary. Then, she underwent a total hysterectomy, contralateral salpingo-oophorectomy and omentectomy without adjuvant treatment.

**Conclusion:**

Optimal cytoreduction has been associated with a statistically significant improvement in survival for malignant transformation of mature cystic teratoma.

## Introduction

Mature cystic teratoma (MCT) is the most common germ-cell tumor of the ovary. It consists of well-differentiated derivatives of the three germ-cell layers [1]. Malignant transformation is a rare complication of this pathology; it accounts for 1-2% of MCTs and the prognosis of this disease is generally poor [2]. In this article, we report a case of MCT who was admitted to National Institute of Oncology in Rabat (Morocco). The diagnosis was proved by histopathologic analysis.

### Case report

**Figure 1:**
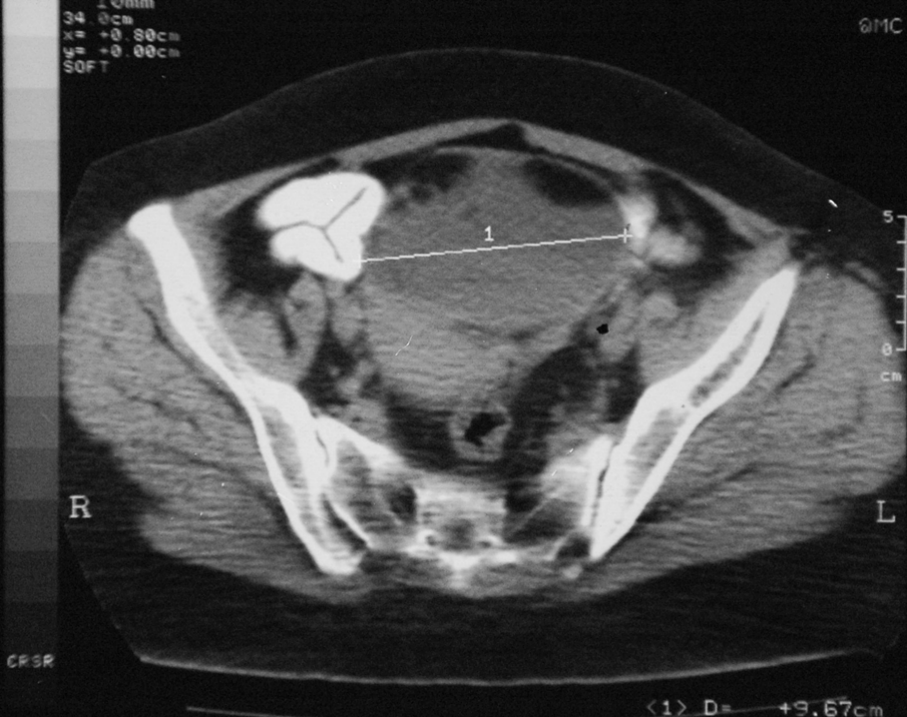
**Abdominal computed tomography scan: a heavily triple tissular mass with greasy and osseous constituent (= 10 cm)**.

**Figure 2:**
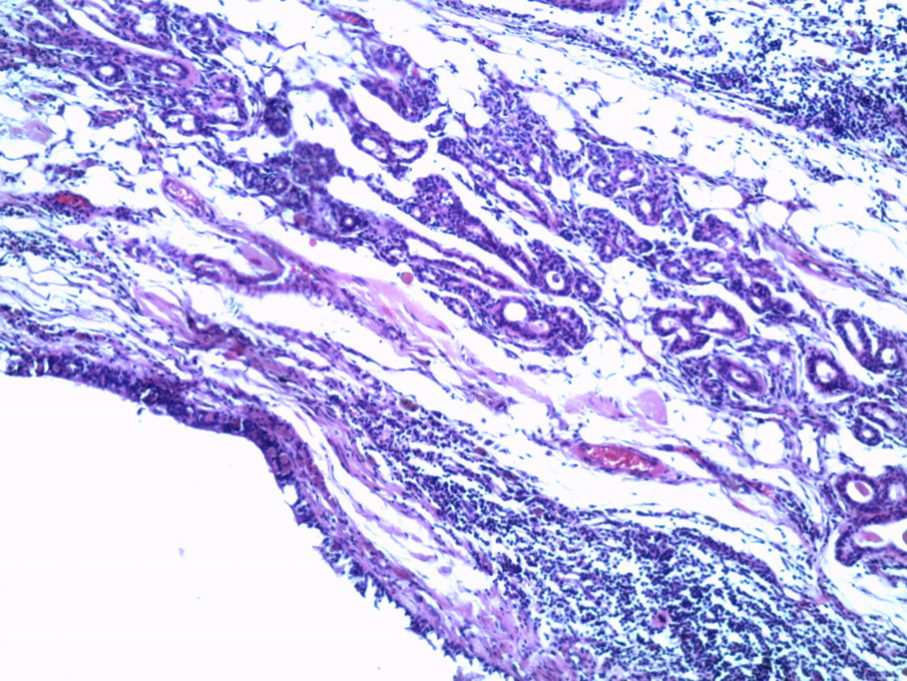
**Mature teratoma (G × 100): respiratory mucosa with adipocytes and smooth muscular fibers**.

**Figure 3:**
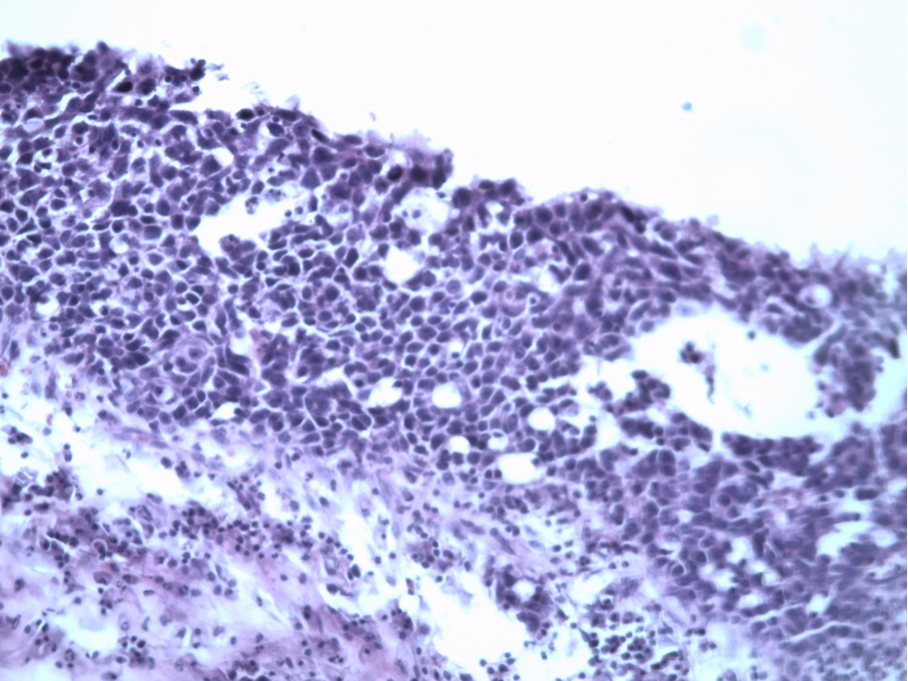
**Squamous cell carcinoma in situ (G × 300): Atypical cells on all the epithelial height with an architectural disorganization**. The basal membrane is intact and the chorion is free.

A 62 year old woman was admitted to a gynecology clinic for adnexal mass which was suspected at first to be a MCT. She had only an abdomino-pelvic pain. Abdominal computed tomography scan revealed a heavily triple tissular mass with greasy and osseous constituent (= 10 cm) (Figure 1). The serum tumour markers (ßHCG, AFP and LDH) were normal. The patient underwent a laparotomy who revealed a voluminous abdomino-pelvic mass. She underwent a left salpingo-oophorectomy only. The histopathologic analysis revealed a squamous cell carcinoma in situ arising in mature cystic teratoma of the ovary (Figure 2 and 3).

Then she was referred to our institution for treatment. Pelvic examination, thoraco-abdominal computed tomography scan and the serum tumour markers (ßHCG, AFP, LDH and CA125) were normal. She underwent a total hysterectomy, contralateral salpingo-oophorectomy and omentectomy.

The histological examination found a mature cystic teratoma in the contralateral ovary without malignant transformation. Adjuvant treatment was not planned.

## Discussion

Ovarian germ-cell tumours account for around 20-25% of ovarian neoplasms and 5% of cancers of the ovary [3]. Mature cystic teratoma (MCT) is the most common ovarian germ cell tumor (10-20% of all ovarian tumors) [1]. Malignant transformation of mature cystic teratomas is very rare (1-2%), with squamous cell carcinoma being the most common type [4]. Pure squamous cell carcinoma in situ arising in an ovarian cystic teratoma is extremely rare [5]. We have only 5 cases from 1976 to 2005 [6].

In our case, it's a squamous cell carcinoma in situ arising in ovarian mature cystic teratoma. In most of the series, the median age at diagnosis of malignant transformation of MCT was 54-61.5 years [7] and the most common symptoms were abdominal pain, palpable mass and abdominal distension; but some people may be asymptomatic at diagnosis [8]. MCT with diameter > 10 cm is associated with increased risk of malignancy in some studies [9]. In our case, the tumor size was 10 cm. Most of studies have found that MCT has a poor prognosis. Early stage and optimal cytoreductive surgery are reported to be good prognostic factors [10].

Due to the relative rarity of the squamous cell carcinoma in situ arising in MCT, there is no uniform consensus regarding treatment. However, the treatment for many authors consist to a complete tumor excision. Adjuvant chemotherapy or radiotherapy is not helpful in improving survival [4-6].

In the review of the literature from 1976 through to 2005, the optimal debulking rate for carcinoma in situ was 100% and the 5-year survival rate for this disease was 100% [6].

## Conclusion

Squamous-cell carcinoma in situ arising in a mature cystic teratoma is an unusual disorder. Because of the rarity of this disease, there is no therapeutic standard at the moment. However, the prognosis seems highly dependent on complete surgical debulking.

## Abbreviations

MCT: Mature cystic teratoma; ßHCG: ß-human gonadotropin; AFP: a-fetoprotein; LDH: lactate dehydrogenase

## Competing interests

The authors declare that they have no competing interests.

## Consent statement

written informed consent was obtained from the patient for publication of this case report and accompanying images.

## Authors' contributions

FAZ: participated in the care of the patient and wrote the article. SO: participated in the care of the patient. SB: participated in the writing of article. MR and LG: realized the histopathologic analysis. HM: participated in the writing of article. HE: Validated content and form of the article. All authors read and approved the final manuscript.
